# Low-Intensity Sonoporation-Induced Intracellular Signalling of Pancreatic Cancer Cells, Fibroblasts and Endothelial Cells

**DOI:** 10.3390/pharmaceutics12111058

**Published:** 2020-11-06

**Authors:** Ragnhild Haugse, Anika Langer, Elisa Thodesen Murvold, Daniela Elena Costea, Bjørn Tore Gjertsen, Odd Helge Gilja, Spiros Kotopoulis, Gorka Ruiz de Garibay, Emmet McCormack

**Affiliations:** 1 Centre for Pharmacy, Department of Clinical Science, The University of Bergen, Jonas Lies vei 65, 5021 Bergen, Norway; ragnhild.haugse@uib.no; 2 Department of Quality and Development, Hospital Pharmacies Enterprise in Western Norway, Møllendalsbakken 9, 5021 Bergen, Norway; 3 Centre for Cancer Biomarkers CCBIO, Department of Clinical Science, The University of Bergen, Jonas Lies vei 65, 5021 Bergen, Norway; Anika.Langer@uib.no (A.L.); Daniela.Costea@uib.no (D.E.C.); bjorn.gjertsen@uib.no (B.T.G.); gorka.garibay@uib.no (G.R.d.G.); 4 KinN Therapeutics AS, Jonas Lies vei 91B, 5021 Bergen, Norway; ethode12@gmail.com; 5 Department of Clinical Medicine, The University of Bergen, Jonas Lies vei 65, 5021 Bergen, Norway; Odd.Gilja@uib.no (O.H.G.); spiros.kotopoulis@uib.no (S.K.); 6 Department of Internal Medicine, Hematology Section, Haukeland University Hospital, Jonas Lies vei 65, 5021 Bergen, Norway; 7 National Centre for Ultrasound in Gastroenterology, Haukeland University Hospital, Jonas Lies vei 65, 5021 Bergen, Norway; 8 EXACT Therapeutics AS, Ullernchausseen 64, 0379 Oslo, Norway; 9 Department of Clinical Science, The University of Bergen, Jonas Lies vei 65, 5021 Bergen, Norway

**Keywords:** sonoporation, microbubbles, ultrasound, intracellular signaling, phosphorylation, ultrasound contrast agents, drug delivery, cellular stress, pancreatic cancer, tumour microenvironment

## Abstract

The use of ultrasound (US) and microbubbles (MB), usually referred to as sonoporation, has great potential to increase the efficacy of chemotherapy. However, the molecular mechanisms that mediate sonoporation response are not well-known, and recent research suggests that cell stress induced by US + MBs may contribute to the treatment benefit. Furthermore, there is a growing understanding that the effects of US + MBs are beyond only the cancer cells and involves the tumour vasculature and microenvironment. We treated pancreatic cancer cells (MIA PaCa-2) and stromal cells, fibroblasts (BJ) and human umbilical vein endothelial cells (HUVECs), with US ± MB, and investigated the extent of uptake of cell impermeable dye (calcein, by flow cytometry), viability (cell count, Annexin/PI and WST-1 assays) and activation of a number of key proteins in important intracellular signalling pathways immediately and 2 h after sonoporation (phospho flow cytometry). Different cell types responded differently to US ± MBs in all these aspects. In general, sonoporation induces immediate, transient activation of MAP-kinases (p38, ERK1/2), and an increase in phosphorylation of ribosomal protein S6 together with dephosphorylation of 4E-BP1. The sonoporation stress-response resembles cellular responses to electroporation and pore-forming toxins in membrane repair and restoring cellular homeostasis, and may be exploited therapeutically. The stromal cells were more sensitive to sonoporation than tumoural cells, and further efforts in optimising sonoporation-enhanced therapy should be targeted at the microenvironment.

## 1. Introduction

The use of ultrasound (US) and microbubbles (MB) in combination with chemotherapy to increase the efficacy of cancer therapy has gained interest in the last 20 years. The term “sonoporation”, is often used to describe this phenomenon. The term describes the formation of pores that occur when cells come into contact with MBs oscillating in an US field [1], hypothesised to enhance uptake for co-administered chemotherapeutics [2]. However, there is still no consensus on what the exact mechanisms underlying US + MBs enhanced cancer therapy are. Furthermore, the cellular stress induced by US + MBs themselves has been proposed to contribute to the anti-cancer effects [3,4].

Despite the insufficient mechanistic understanding, substantial in vitro [5,6,7,8,9,10,11,12,13] and in vivo research [5,6,7,8,9,14,15,16,17] has shown that US + MBs-enhanced cancer therapy is beneficial for cancer therapy. In 2016, results from the first Phase 1 human clinical trial using US + MBs in combination with chemotherapy to treat pancreatic ductal adenocarcinoma (PDAC) were published [18]. PDAC is a deadly cancer, with less than an 8% five-year overall survival [19], which requires better treatment options. The clinical trial demonstrated that the use of US + MBs is safe, and a secondary endpoint indicated that sonoporation + chemotherapy (gemcitabine) may increase survival of patients. PDAC is characterised by extensive desmoplastic stroma, thought to originate from cancer-associated fibroblasts (CAFs), and a complex hypoxic tumour microenvironment, which are major contributors to resistance to chemotherapy [19,20]. Some hypothesised effects of sonoporation on the tumour microenvironment are increased drug extravasation from the blood vessels or destruction of tumour vasculature [2], but the relevance of microenvironmental effects for the clinical efficacy of sonoporation remains largely unknown.

In vitro sonoporation research has typically focused on cancer cell lines, evaluating the sonoporation efficacy either by uptake of cell impermeable dyes [5,8,9,21] and/or by evaluation of the viability of cells exposed to sonoporation [21] or sonoporation in combination with drugs [5,6,7,8,9,22]. Based on the fact that MBs are usually injected into the vasculature, in vitro studies have also addressed the effects of sonoporation on endothelial cells, showing increased permeability both in the cellular membrane [23,24] and interendothelial openings between cells [25,26,27,28].

Our previous study [4] using leukemic cells as a model system of cancer compared to healthy peripheral blood cells demonstrated that different cell types have different sensitivities to sonoporation in terms of molecular uptake, effect on viability and intracellular signalling. This was a simplified system compared to solid tumours, which consist of the complex tumour microenvironment with stromal cells, immune cells, fibroblasts, blood vessels and extracellular matrix (ECM) in addition to, and supporting, the cancer cells [29]. The observed difference in sonoporation sensitivity raises the question of whether the cell types within a solid tumour may also respond differently, meaning that treatment of the cancer cells might not be the most important sonoporation effect in the enhancement of chemotherapeutic efficacy. Investigations on the cellular responses to US + MBs should, therefore, be carried out in cell types relevant for the tumour microenvironment.

In this study, we aim to improve our understanding of how sonoporation may affect pancreatic cancer by evaluating the effects on the uptake of cell impermeable dye, viability and intracellular signalling response in PDAC, endothelial and fibroblast cell types. The results from this study will help expand our understanding of how the different cell types respond to sonoporation, which is needed for the development of better in vitro and in vivo models for sonoporation, the optimisation of sonoporation parameters and the choice of drugs. In fact, the results indicate that the cancer cells are not the most sensitive to sonoporation in terms of uptake of cell-impermeable molecule, reduction in viability or intracellular signalling response (phosphorylation of p38, ERK1/2, ribosomal protein S6 and 4E-BP1), further suggesting that cells in the tumour microenvironment may be relevant for sonoporation efficacy.

## 2. Materials and Methods

### 2.1. Chemicals

All chemicals were purchased from Merck KGaA (Darmstadt, Germany) unless otherwise stated.

### 2.2. Maintenance Cell Culture

Pancreatic ductal adenocarcinoma cell line MIA PaCa-2 (ATCC^®^ CRM-CRL-1420^TM^, kindly donated by Professor Anders Molven, University of Bergen, Bergen, Norway) was cultured in high-glucose Dulbecco’s modified Eagle’s medium (DMEM #5671) supplemented with 10% foetal bovine serum (FBS), 2% L-glutamine, 1 mM sodium pyruvate and 2.5% horse serum. Human foreskin fibroblasts (BJ, ATCC^®^ CRL-2522^TM^, kindly donated by professor Donald Gullberg, University of Bergen, Bergen, Norway) were cultured in high-glucose DMEM #5671 supplemented with 10% FBS, 1% L-glutamine and 50 U/mL Penicillin/50 U/mL Streptomycin. Single-donor lot human umbilical vein endothelial cells (HUVECs) (Cat#CC-2517, Lonza, Basel, Switzerland, kindly donated by Prof. Jim Lorens, University of Bergen, Bergen, Norway) were cultured in EGM^TM^-2 medium supplemented with EGM^TM^-2 Endothelial Cell Growth Medium-2 BulletKit^TM^ (Lonza). Cell culture medium was changed on the HUVECs every second day according to suppliers’ recommendations, except when cultured in Petaka G3 LOT^®^ (Celartia Ltd., Powell, OH, USA) [c.f. Section 2.4]. HUV-EC-Cs (ATCC^®^ CRL-1730^TM^) were cultured in F12-K medium (ATCC, Manassas, VA, USA) supplemented with endothelial growth supplement from bovine neural tissue (#E2759) and 10% FBS. All cells were cultured in a 5% CO_2_ humidified atmosphere at 37 °C.

### 2.3. Isolation of Cancer-Associated Fibroblasts (CAFs)

CAFs were isolated from cancer tissue biopsies of PDAC patients with primary lesions after informed consent, and in accordance with the Declaration of Helsinki and approval by the Ethics Committee (REK 2013/1772). In brief, tissues were collected and washed in Dulbecco’s modified Eagle’s medium (DMEM #6429) supplemented with 2% antibiotic-antimycotic (AB/AM), 100 U/mL penicillin, 100 µg/mL Streptomycin and 25 ng/mL Amphotericin B (Thermo Fischer Scientific, Waltham, MA, USA). Bleeding and necrotic areas of the tissues were cut out using a sterile scalpel and washed thoroughly. Tumour tissues were then cut into approximately 2–4 mm^2^ tissue bits and then transferred onto a 10 cm culture dish, slightly air-dried (approximately 2 min) to allow explants to attach on the growth surface of the dish, and incubated in FAD medium (DMEM/Nutrient Mixture F-12 Ham supplemented with 10% FBS, 0.4 mg/mL hydrocortisone, 1% Insulin-Transferrin-Selenium, 50 mg/mL L-ascorbic acid, 10 ng/mL epidermal growth factor and 1% AB/AM). Explants with an outgrowth of cells with fibroblast morphology were trypsinized in a clonal ring placed around the respective explant and fixed on the bottom of the dish with sterile vaseline and placed in 6-well plates. Isolated fibroblasts were further characterised for lineage-specific markers: ESA, CD31, CD45 and CD140b for epithelial, endothelial, blood-borne and mesenchymal origin, respectively. CAFs were further cultured in DMEM (#6429) supplemented with 10% FBS and without AB/AM.

### 2.4. Cell Culture for Experiments

**Figure 1:**
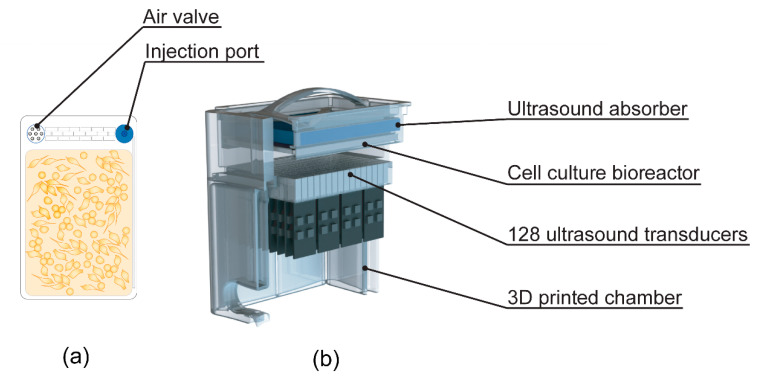
(**a**) Drawing of cell culture bioreactor (Petaka) used for culturing of cells prior to ultrasound (US) treatment; (**b**) cutaway of custom-made US treatment chamber used for US treatment of cells (adapted from [22], Pharmaceutics, 2020).

Three days prior to experiments, 26 mL of cell suspension was injected in Petaka G3 LOT^®^ (low oxygen transport) cell culture chambers (Figure 1a referred to as Petaka herein). A suitable number of cells were injected to achieve confluency at the time of US/MB exposure. To avoid the influx of air, the air valve on the Petakas was sealed with tape, and they were cultured in the horizontal position for a minimum of 24 h to allow for cells to adhere to the plastic surface. The Petaka chambers are designed for cell culture within a limited gas exchange and a gradually decreasing oxygen concentration [30], which is closer to pO_2_ (“physioxia”) found in living tissue compared to the “normoxic” conditions at atmospheric O_2_ pressure commonly used in vitro [31].

### 2.5. Microbubbles

Sonazoid^TM^ (GE Healthcare, Little Chalfont, UK) was reconstituted by adding 2 mL NaCl 9 mg/mL (B Braun, Melsungen, Germany) and gently agitated for 30 s. MBs were aspirated via a 19 G needle and transferred to an eppendorf tube. A 19 G venting needle was used to avoid a pressure drop in the vial. To ensure that the reconstituted bubbles were stable, Sonazoid™ bubbles were used within 1 h of reconstitution. A 60 µL volume of reconstituted Sonazoid™ (1.2 × 10^9^ bubbles/mL) was diluted in NaCl 9 mg/mL to a total volume of 1 mL and injected into the Petaka immediately prior to US exposure, giving a concentration of 2.8 × 10^6^ bubbles/mL in the Petaka. In the untreated sample, and US alone controls, 1 mL of NaCl 9 mg/mL was added to Petaka. The Petakas were gently rolled in all directions to ensure a homogenous distribution of MBs and NaCl.

### 2.6. In Vitro Treatment with Ultrasound and Microbubbles

The cells were exposed to US using a custom-made US treatment chamber (Figure 1), based on a previous design [32] and previously used in [22], suited for US exposure of adherent cells when cultured in Petakas.

The US system consisted of 128, 9 × 6 mm PZ26 elements firing upwards as a plane-wave into the Petaka, ensuring US treatment of the entire cell-covered surface in the Petaka. The US transducers were driven by a custom Open Ultrasound system (Lecoeur Electronique, Chuelles, France). The acoustic field had been calibrated in three axes using a 200 µm needle hydrophone (Precision acoustics Ltd., Dorset, UK) in the fully assembled US chamber, and the Petaka was placed at the acoustic focus.

Before US treatment, air pockets were removed from the Petaka. To ensure that the floating MBs would come into contact with the cells, the Petaka was placed in the US treatment chamber (Figure 1) with the cell-covered side on top, closest to the US absorber. A low-Mechanical Index (MI) (<0.01) B-mode scan was performed before treatment with US ± MB to detect air pockets in the US bath between the water medium and the Petaka. Air pockets, if present, were removed before US treatment. The US conditions used were based on a previous study [22] and referred to as “Medium US” or “High US” (see Table 1 for details). As untreated control, a Petaka, containing cells but no MBs, was placed in the US treatment chamber for 5 min without application of US. All experiments were performed in triplicate at minimum, except CAFs, where sonoporation experiments were only performed once and only treated with “High US”, due to limited material availability. Imaging of cells was performed immediately after sonoporation with 10× magnification using a Nikon Eclipse E200 microscope equipped with a Lumenera Infinity 1 camera.

### 2.7. Calcein Uptake

To assess if the cell membrane had been permeabilised, a nontoxic cell-impermeable fluorescent dye, calcein, was added during treatment with US ± MB. A 50 mg/mL calcein stock solution in 1 M NaOH was kept at 2–8 °C protected from light. Immediately prior to treatment with US ± MB, calcein was mixed with NaCl 9 mg/mL ± MBs in the 1 mL that was injected into the Petaka. A concentration of 6 μM calcein was used in all experiments.

After treatment with US ± MB, the cells were incubated for 1 h to allow for cell membrane pores to re-seal [4,33], flushed twice with phosphate-buffered saline (PBS), detached using trypsin-EDTA 0.05% and harvested from the Petaka. Following centrifugation and resuspension in PBS, the cells were analysed by flow cytometry on an Accuri C6 flow cytometer (BDBioscience, Franklin Lakes, NJ, USA). Data collected from Acurri C6 were gated in FlowJo^®^, and the uptake of calcein was measured as a percentage of calcein-positive cells. The gating strategy is shown in Supplementary Figure S1. The median fluorescence intensity (MFI) of the cells in the calcein-positive population was also recorded.

### 2.8. Viability Analysis: Apoptosis and Cell Death

Cells were treated with US ± MBs and cultured in Petaka placed in a 5% CO_2_ humidified atmosphere at 37 °C for 24 h. Following incubation, the medium from the Petaka was collected, cells were flushed with phosphate-buffered saline (PBS) and the cells were detached from the plastic using Accutase^®^. Harvested medium and cell suspension were combined, and cells were counted using a haemocytometer (later referred to as cell count 24 h). Trypan Blue^®^ (Thermo Fischer Scientific, Waltham, MA, USA) was added to identify dead cells. Apoptosis was assessed by staining with Annexin V antibody and propidium iodide (PI) using Dead cell Apoptosis kit with Annexin V Alexa Fluor^TM^ 488 and Propidium Iodide *(PI)* (Invitrogen, Catalog #V12341, Thermo Fischer Scientific, Waltham, MA, USA). The assay was performed in accordance with the manufacturer’s protocol with two exceptions: Half the concentration of Annexin V antibody and of PI were used, based on titration, and PI was added shortly before flow cytometry analysis. Data were collected on an Acurri C6 flow cytometer and gated in FlowJo^®^. The gating strategy is shown in Figure S2.

To assess cell loss during US exposure (e.g., due to cell detachment or destruction), the cell count per Petaka was counted using a haemocytometer at 0 h (as described in Section 2.10). As mentioned above, cells were also counted after 24 h to assess sonoporation effects on viability. CAFs could not be reliably counted in the haemocytometer, due to low cell concentration. CAFs were counted 24 h after US exposure by analysis of a fixed volume of cell suspension (300 μL) using an Accuri C6 flow cytometer.

### 2.9. Viability Analysis: Growth Potential and Metabolic Activity

Cells harvested from the Petaka 24 h post-sonoporation were used to assess their proliferative capacity after re-seeding. Live cells (Trypan blue^®^ negative) were seeded on 96-well cell culture plates (MIA PaCa-2: 3000 cells, fibroblasts: 6000 cells, HUVEC: 3000 cells), and their metabolic activity was assessed after 24, 48 and 72 h by addition of WST-1 reagent (Roche Diagnostics GmbH, Mannheim, Germany). WST-1 was added 2 h before detection on a multiwell spectrophotometer in accordance to the manufacturer’s protocol. Live cells were also seeded on 24-well plates (MIA PaCa-2: 30,000 cells, fibroblasts: 60,000 cells, HUVEC: 30,000 cells), and the cells were detached using Accutase^®^, diluted in cell culture medium, and counted after 24, 48 and 72 h using a haemocytometer.

### 2.10. Sample Preparation for Phosphospecific Flow Cytometry

To investigate changes in intracellular signalling events, cells were harvested from separate Petakas as soon as possible after sonoporation and after 2 h of incubation. Timepoints were selected based on a previous study [4]. Cells cultured in separate Petakas were treated with 1 μM A23187 (calcium ionophore) + 100 nM phorbol myristate acetate (PMA: PKC activator) for 30 min as positive controls for intracellular signalling. Cells were detached from the Petaka using the cold trypsin method [34,35], i.e. with ice-cold 2.5% trypsin not containing ethylenediaminetetraacetic acid (EDTA). Prior to cell detachment, the medium was harvested, the Petaka was flushed once with ice-cold PBS, and the collected cell culture medium and PBS were placed on ice during detachment of cells. Ice-cold 2.5% trypsin was added to the Petaka and subsequently placed on ice during the detachment time. In all experiments, the cell detachment on ice started within 1–3 min after US exposure. Cell detachment time varied between cell lines, as shown in Figure S3. Cell culture medium, PBS, and cells were collected and fixed by adding 16% paraformaldehyde (PFA, Alfa Aesar, Haverhill, MA, USA) directly to yield a final concentration of 2%, incubated for 15 min at room temperature and permeabilised by adding ice-cold methanol [4,36]. Before addition of PFA, a sample was taken for cell count (later referred to as cell count 0 h) and counted using a haemocytometer.

### 2.11. Barcoding

To reduce antibody staining variability, the samples were barcode-stained. The six individual cell samples were stained with unique signatures of succinimidylesters of Pacific Blue and Pacific Orange (barcoding) for multiplex flow cytometry [37]. After barcode-staining, the samples were washed in PBS containing 1% bovine serum albumin and 2 mM EDTA, then pooled prior to antibody staining. A graphical depiction of barcoding/sample preparation is shown in Figure S4: One barcode represents all five samples from one timepoint in each experiment (Untreated cells, Medium US, High US, Medium US + MBs, High US + MBs) and a positive control. Pooled cells were split into different tubes and each tube was stained with an antibody panel. Each panel consisted of a combination of two antibodies conjugated to either Alexa Fluor^®^ 488 or 647 (Table S1). The panels of markers were based on our previous study [4]: Mitogen-activated protein kinase (MAPK; p38 and extracellular regulated kinase 1/2 (ERK1/2), cAMP response element-binding element (CREB), protein kinase A (PKA), signal transducer and activator of transcription 3 (STAT3; 727 epitope), phosphoinositide 3-kinase (PI3K), Akt and mammalian target of rapamycin (mTOR) pathway proteins (ribosomal protein S6) and eukaryotic translation initiation factor 4E-binding protein (4E-BP1). The panel was extended to include focal adhesion kinase (FAK) and Src, based on studies on mechanotransduction in response to US [38,39,40]. Samples were analysed on an LSR Fortessa flow cytometer (BD Bioscience, Franklin Lakes, NJ, USA).

### 2.12. Data Analysis of Phosphospecific Flow Cytometry

Data collected on the LSR Fortessa were compensated, gated and de-barcoded in DIVA software. The gating strategy is shown in Figure S5. Analysis of median fluorescence intensity (MFI) was performed in Cytobank (Cytobank Inc., Santa Clara, CA, USA). The MFI of each sample was corrected for autofluorescence of the cells and the barcode staining by subtraction of MFI of the corresponding barcoded cells unstained with antibody. The arcsinh ratio (arcsinh (treated/5)−arcsinh (control/5)) was calculated in Microsoft Excel to depict changes in phosphorylation.

### 2.13. Statistical Analysis

Statistical comparisons were performed in GraphPad Prism 8 (San Diego, CA, USA). A Shapiro-Wilk normality test was performed on all datasets to determine if the data were normally distributed. As over 95% of the datasets passed the normality tests, a repeated-measures one-way Analysis of Variance (ANOVA) with Holm–Sidak’s multiple comparison test of the sample MFI versus the untreated samples was used. In addition, an ordinary ANOVA was used for statistical comparisons of calcein uptake and viability (treated cells versus untreated cells; medium US + MBs vs. high US + MBs only for % calcein-positive cells). Significance level was set at *p*-value 0.05.

## 3. Results

### 3.1. Direct Effects of Sonoporation: Uptake of Cell Impermeable Dye and Cell Lysis (Cell Count)

**Figure 2:**
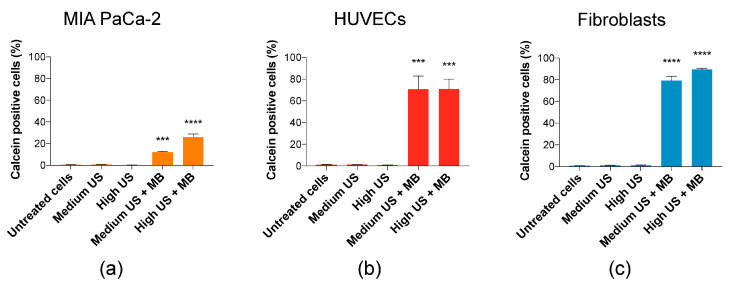
The percentage of cells taking up calcein. Addition of MBs was necessary for increased uptake in cells. (**a**) In MIA PaCa-2, increased US intensity increased the percentage of calcein-positive cells. (**b**) In human umbilical vein endothelial cells (HUVECs), increased US intensity was not important for percentage of calcein-positive cells. (**c**) In fibroblasts, increased US intensity resulted in a small increase in percentage of calcein-positive cells. Mean ± SEM; *** *p* < 0.001, **** *p* < 0.0001 (treated vs. untreated cells).

In all cell types, uptake of calcein was only observed when MBs were added, suggesting that increased uptake only occurs after sonoporation. The lowest uptake was observed in MIA PaCa-2, 12% of cells at Medium US + MBs and 25% at High US + MBs (Figure 2a). In HUVECs, the percentage of cells taking up calcein was high (70%) at both Medium and High US + MBs (Figure 2b). The percentage of cells taking up calcein was even higher in fibroblasts, and increased with US intensity from Medium US (79%) to High US (90%) (Figure 2c). The increase in US parameters from medium US to high US had a significant effect on uptake in MIA PaCa-2 and fibroblasts (*p* < 0.001 and *p* < 0.01, respectively (statistic not displayed in Figure 2)).

**Figure 3:**
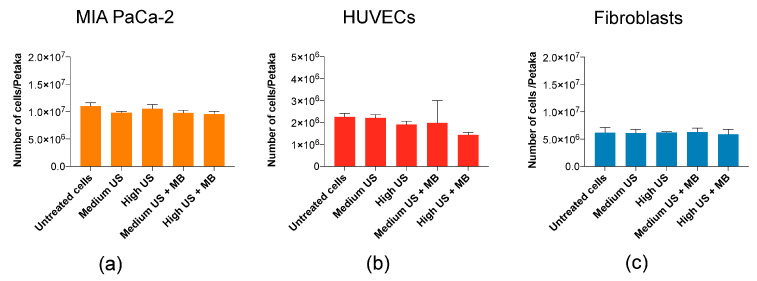
Cell count immediately (0 h) after sonoporation. Cell count (0 h) indicated no mechanical destruction of cells in (**a**) MIA PaCa-2 and (**c**) fibroblasts, while a minor and nonsignificant reduction was observed in (**b**) HUVECs. Mean ± SEM.

The sonoporation parameters used in this study did not induce a reduction of cells (cell lysis) in any of the cell lines used (Figure 3).

### 3.2. Cellular Viability upon Sonoporation

**Figure 4:**
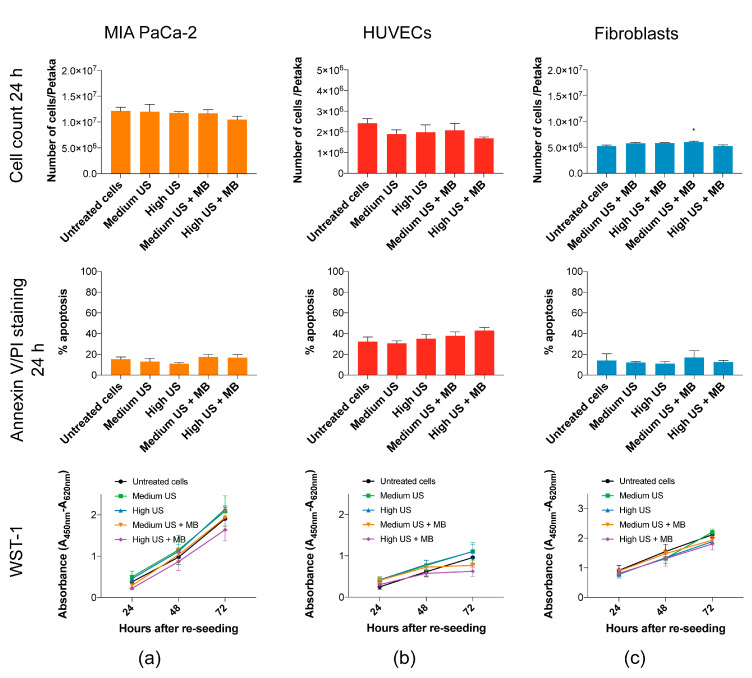
Viability of cells harvested after 24 h of incubation in Petaka. (**a**) No significant reduction in the cell count or increase in apoptotic AnnexinV/PI-stained cells of MIA PaCa-2 was observed. The metabolic activity (WST-1) after re-seeding was not affected by US + MB. (**b**) Cell count 24 h post-sonoporation of HUVECs is slightly, but not significantly, decreased in treated samples. Percentage of apoptotic cells 24 h post-sonoporation by AnnexinV/PI staining was increased, and also not statistically significant. No significant change was observed in the metabolic activity (WST-1) of the cells after re-seeding of the cells, although some reduction in metabolic activity was observed in cells exposed to US + MBs. (**c**) No significant reduction in the cell count, increase in apoptotic AnnexinV/PI stained cells or reduced metabolic activity (WST-1) after re-seeding of fibroblasts was observed. Mean ± SEM. * *p* < 0.05 (treated vs. untreated cells).

Sonoporation may induce apoptosis [41,42,43,44] and reduce proliferation of cells [4]. Sonoporation at the US intensities in this study had minimal negative effects on the viability of MIA PaCa-2, HUVECs and fibroblasts. In MIA PaCa-2, no increase in percentage of apoptotic cells were observed by Annexin/PI staining (Figure 4a), although a small increase in dead cells at High US (*p* < 0.05) was observed by Trypan Blue staining in samples taken directly after collecting cells and medium (Figure S6). In HUVECs, apoptosis measured by Annexin V/PI staining increased with increasing US + MBs, but this was not statistically significant (Figure 4b). The uptake of Trypan Blue in HUVECs at these parameters was significantly different from untreated cells (*p* < 0.01) (Figure S6). In fibroblasts, a very small, but statistically significant (*p* < 0.05), increase in cell count was observed after 24 h of culturing after sonoporation with Medium US + MBs (Figure 4c).

The long-term viability after the re-seeding of cells was not significantly reduced in any of the cell types (WST-1 in Figure 4, confirmed by cell counts in Figure S6). In HUVECs, there is a trend towards reduced cell metabolic activity and growth after 48 h in cells treated with US + MBs, although this was not significant.

### 3.3. Sonoporation Induced Changes in Intracellular Signalling

**Figure 5:**
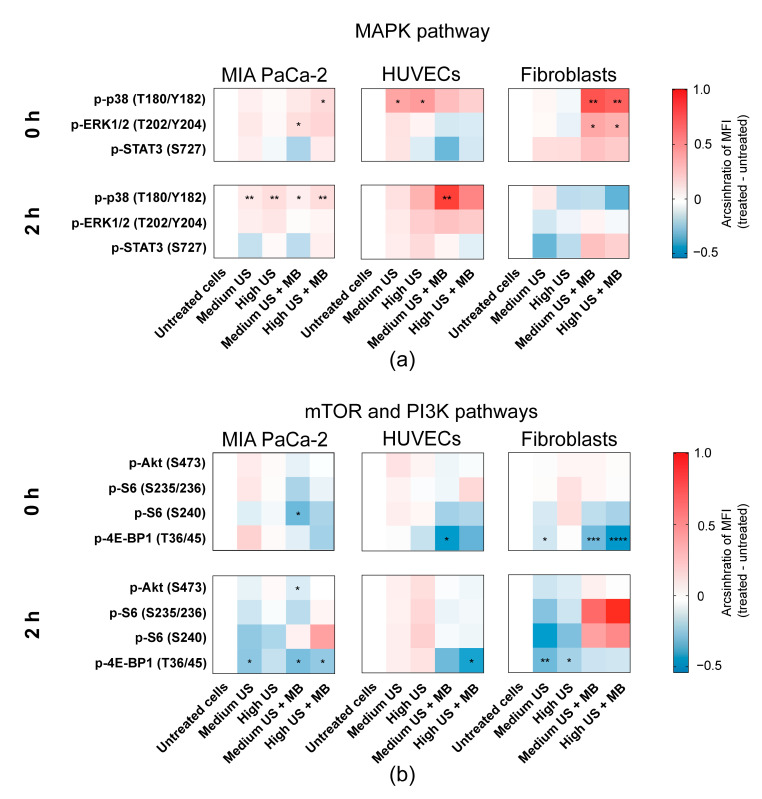
Intracellular signalling induced by sonoporation. Heatmaps displaying changes in phosphorylation status (shown as arcsinh ratio) of the chosen range of proteins in response to treatment with US with and without Sonazoid^TM^ MBs. Phosphorylation status was detected immediately (0 h) and 2 h post-sonoporation. Different phosphorylation profiles were observed in the (**a**) MAP-kinase pathway (p38, ERK1/2 and downstream target STAT3 S727), and (**b**) mTOR (ribosomal protein S6 and 4E-BP1) and PI3K pathways (Akt). Mean ± SEM. * *p* < 0.05, ** *p* < 0.01, *** *p* < 0.001, **** *p* < 0.0001 (treated vs. untreated cells).

Sonoporation induced phosphorylation of MAP-kinases p38 T180/Y182 and ERK1/2 T202/Y204 in all three cell types, but with different magnitudes and timings (Figure 5a). The most pronounced activation was observed in fibroblasts immediately after sonoporation, while the activation on MIA PaCa-2 and HUVECs was weaker or delayed. In MIA PaCa-2, an immediate, weak activation of both p38 T180/Y182 (Medium and High US + MBs; *p* = 0.15 (ns) and *p* < 0.05, respectively) and ERK1/2 T202/Y204 (Medium and High US + MBs, *p* < 0.05 and *p* = 0.15 (ns), respectively) was observed in cells treated with the combination of US + MBs (Figure 5a). p38 was still significantly activated 2 h after sonoporation, but very weakly. In HUVECs, phosphorylation of p38 T180/Y182 was moderately increased in cells treated with US both with and without MBs immediately after sonoporation, although this effect was only significant in cells treated without MBs (*p* < 0.05) (Figure 5a). After 2 h, p38 T180/Y182 phosphorylation was still elevated, but lower than that at 0 h, in HUVECs treated without bubbles (ns). In HUVECs treated with US + MBs, phosphorylation of p38 T180/Y182 was further increased 2 h after sonoporation (Medium US and High US + MBs; *p* < 0.01 and *p* = 0.08 (ns), respectively). ERK1/2 T202/Y204 phosphorylation was unchanged in HUVECs immediately after sonoporation, but a small insignificant increase was observed 2 h after sonoporation in cells treated with US + MBs. In fibroblasts, US + MBs induced significant, immediate changes in the phosphorylation level of MAP-kinases p38 T180/Y182 (*p* < 0.05) and ERK1/2 T202/Y204 (*p* < 0.01) In response to both Medium and High US (Figure 5a). Two hours after sonoporation, the phosphorylation level was returned to the basal level. The phosphorylation of downstream target STAT3 S727 was not significantly changed in any of the cell types, although STAT3 S727 was weakly increased immediately and 2 h after sonoporation in fibroblasts.

In the mTOR pathway, 2 h after sonoporation, phosphorylation of ribosomal protein S6 was increased in MIA PaCa-2 at the S240 epitope and in fibroblasts at both the S240 and the S235/236 epitopes (Figure 5b). However, phosphorylation of S6 was not statistically significant and varied between experiments (Figure S7). Unlike the other cell lines, phosphorylation of ribosomal protein S6 was not increased at 2 h in HUVECs.

In general, sonoporation caused dephosphorylation of 4E-BP1 T36/45, particularly when MBs were added (Figure 5b). In MIA PaCa-2, 4E-BP1 T36/45 was significantly dephosphorylated 2 h after sonoporation using Medium US and Medium/High US + MBs (*p* < 0.05). Phosphorylation of 4E-BP1 T36/45 in HUVECs was decreased immediately using Medium US and High US + MBs (*p* < 0.05 and *p* = 0.09 (ns), respectively), and at 2 h after using sonoporation High US + MBs (*p* < 0.05). In fibroblasts, the dephosphorylation of 4E-BP1 T36/45 was most pronounced immediately after sonoporation MB (Medium and High US + MBs; *p* < 0.001 and *p* < 0.0001, respectively). 4E-BP1 T36/45 was also dephosphorylated in the fibroblasts in response to US without MBs, immediately after sonoporation using Medium US (*p* < 0.05) and 2 h after using Medium and High US (*p* < 0.01 and *p* < 0.05, respectively).

The only statistically significant change in phosphorylation of Akt S473 was observed in MIA PaCa-2, 2 h after sonoporation, but this effect was very small (*p* < 0.05) (Figure 5b). Changes in phosphorylation of CREB, PKA, Src and FAK, together with all experiments with CAFs, were nonsignificant, and are presented in Supplementary data (Figures S8–S10).

### 3.4. Induction of Apoptosis by Inhibition of MEK/ERK in Combination with Sonoporation

**Figure 6:**
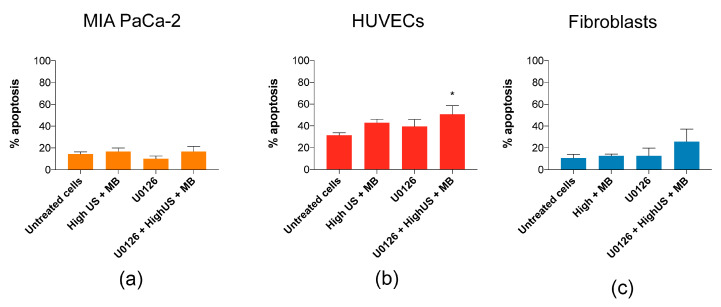
Inhibition of MEK/ERK with U0126 in combination with high US + MBs increased the percentage of apoptotic (**b**) HUVECs (*p* < 0.05) and (**c**) fibroblasts, but not (**a**) MIA PaCa-2. Mean ± SEM, * *p* < 0.05 (treated vs. untreated cells).

The role of the ERK1/2 activation in response to sonoporation is not yet known, but has been shown to be important for cellular recovery in cells exposed to pore-forming toxins through an intracellular mechanism involving p38 and ERK1/2 (similar to observations in fibroblasts in Figure 5) [45]. Similar to Cabezas et al. [45], we treated cells with MEK/ERK inhibitor U0126 prior to sonoporation, which increased the percentage of apoptotic fibroblasts (from 13% to 26%) and the percentage of apoptotic HUVECs from 43% to 51% (Figure 6), but not at all in MIA PaCa-2. However, the increases were only significant in HUVECs (*p* < 0.05).

## 4. Discussion

### 4.1. Sensitivity to Sonoporation

In this work, we aimed to compare the sonoporation efficacy and intracellular signalling responses to sonoporation in a selection of cell lines representative of the cellular diversity present in solid tumours. The uptake of calcein and other cell-impermeable dyes is commonly used in sonoporation research as a measure of cell permeabilization and successful drug uptake [5,8,41,46,47,48,49]. The dye uptake may be measured both as a percentage of stained cells and sonoporation efficiency (amount of molecules taken up, measured as fluorescence intensity). In previous studies, the percentage of stained cells and sonoporation efficiency followed the same trend [4,33], but contrary to expectations, opposite trends were observed in percentage and efficiency (MFI) between the cell types in this study (Figure 2 and Supplemental Figure S11). For the purpose of this discussion, we have focussed on percentage calcein-stained cells (Figure 2), which is the most commonly used measure. Furthermore, this measurement followed similar trends as the observed changes in intracellular signalling.

Interestingly, the cancerous MIA PaCa2 cells experienced a considerably lower percentage of calcein-positive cells in both the medium and high US settings (12%/25%) compared to HUVECs (70%/70%) and fibroblasts (79%/90%). Whilst high US + MB was shown to be most efficient in sonoporation, the higher ultrasound intensity might lead to cell damage. However, the low-intensity US regimen used in this study did not have a major impact on cell viability. It has previously been concluded that cancerous cells may be more sensitive to US ± MB, both in terms of viability [50,51], or viability and uptake of cell-impermeable dye [4], but the current results indicate that sonoporation efficacy is not really a question of healthy versus cancer cells. In addition, different PDAC cancer cell lines have different sensitivities to sonoporation, as shown by Bjånes et al. (Supplementary Data) [22].

Sonoporation efficacy has been associated with multiple factors. While larger cells have a greater likelihood of interaction with microbubbles, additional factors might be more relevant [51]. This is supported by our results on sonoporation efficacy in small HUVECs versus larger HUV-EC-C cells (HUVEC cell line) (Figure S12) where the difference in uptake was not significant, and a larger difference was actually observed between HUVECs and MIA PaCa-2 despite their similarity in cell size (Figure S13). Cell membrane stiffness is also proposed to influence sonoporation [52], which should be explored in further studies.

### 4.2. Induction of Sonoporation Signalling

Activation of intracellular signalling follows a similar pattern as observed in our previous study on a leukemic cell line and peripheral mononuclear blood cells (PBMCs) [4], suggesting a general mechanism across cell types and irrespective of differences in the culturing of suspension and adherent cell lines. In both studies, the magnitude of activation follows the trend of calcein uptake (i.e. extent of permeabilisation). The most important pathways involved were activation of the MAP-kinases p38 and ERK1/2 (and CREB, STAT3, Akt in [4]), and activation of either 4E-BP1 or eIF2α [4], and ribosomal protein S6 2 h post-sonoporation. Just as in MIA PaCa-2, the changes in intracellular signalling previously observed were overall weaker in PBMCs [4], where a lower proportion of cells were sonoporated.

No major impact on cell viability was observed in this study, except a small increase in apoptotic fibroblasts and HUVECs when MEK/ERK was inhibited using U0126. The activation of MAP-kinase (p38, ERK) and dephosphorylation of 4E-BP1 in response to sonoporation resembles signalling related to membrane repair following pore formation and osmotic stress in cells exposed to pore-forming toxins [45,53] and electroporation [54,55]. The similarity was most pronounced in fibroblasts, which may explain why viability was not affected in the fibroblasts in spite of the high rate of sonoporation (calcein uptake). The mechanism for repair of sonoporation-induced pores, i.e. repairable sonoporation [56], is still not yet fully known but has also been compared by others to membrane repair in cells exposed to pore-forming toxins [57]. The relationship between pore formation (by sonoporation, pore-forming toxins, electroporation), signalling events and membrane repair requires further evidence in future studies.

Dephosphorylation of 4E-BP1 is most commonly known to suppress protein synthesis through inhibition of cap-dependent protein translation [58,59], which under stressful conditions, may be regulated though the unfolded protein response (UPR) to restore cellular homeostasis [60]. However, the sonoporation-inhibition of 4E-BP1 may itself contribute to the anti-cancer effects of sonoporation. In PDAC therapy, inhibition of 4E-BP1 in cancer-associated fibroblasts was found to repress secretion of proteins involved in chemoresistance, and to improve the efficacy of chemotherapy (gemcitabine) by acting as a stroma-targeted therapy [61].

Together with dephosphorylation of 4E-BP1, sonoporation induced a paradoxical phosphorylation of ribosomal protein S6 in the mTOR signalling pathway. The role of S6 activation in response to sonoporation is not yet known, but we observed this activation to be absent under conditions where cellular viability was decreased by sonoporation in our previous study on leukemic cells too [4]. Typically, activation of ribosomal protein S6 stimulates cap-dependent protein translation downstream of mTOR [62]. The activation may imply that cellular homeostasis is restored, resulting in stimulated protein synthesis, but further studies are required to confirm this.

### 4.3. Limitations of This Study and Future Perspectives

Whilst the effects of sonoporation on cancer cells have been extensively studied and, to some extent, also on HUVECs, the effects on fibroblasts, cancer-associated fibroblasts and other cells of the tumour microenvironment are less known. As these results show that the different cell types respond differently to sonoporation, it is important to include the cells of the tumour microenvironment in future studies. This study was limited by the inclusion of only one cell line for each cell type and only one cancer cell line. Based on differences in sonoporation efficacy between PDAC cell lines [22] differences in magnitude of signalling changes may be anticipated.

The main limitations are that each cell type was studied separately and cultured as a monolayer on a plastic surface with no regulation of liquid flow, temperature or gas saturation. Cells were cultured in Petaka G3 LOT cell culture chambers. Even though these are designed to regulate gas exchange, pO_2_ depends on the cell line and cell counts, and a limitation in these experiments is that pO_2_ was not monitored, as this parameter may affect both the physical properties of microbubbles and viability/responses of the cells.

Recent studies on interendothelial openings between cells [25,26,27] suggest that the effects of sonoporation are beyond the simple formation of pores in cancer cells interacting with bubbles in an US field. However, this was also studied on monolayers of endothelial cells alone. The term “sonopermeation” has been introduced to cover the broader range of effects leading to increased delivery of drugs, including endocytosis, opening of cellular junctions and changes in vessels and the stromal compartment [63], but actual determinations of the role of different cell types and the mechanism of sonoporation require more advanced organoid models or preclinical studies with humanised immune systems and tumours. Furthermore, as the results in all cell lines in this study confirm that MBs are essential for sonoporation, more advanced models are also required to assess if MBs only affect the endothelium or if the reduced viability and opening of junctions between endothelial cells can lead to interaction with the tumour microenvironment and cancer cells.

The majority of these studies have been performed in triplicate due to the complexity of the technical process required to obtain results. This may impact the statistical analysis and potential conclusions that can be drawn from this study. Further work and additional repetitions should be performed to independently validate these results.

## 5. Conclusions

Different cell types respond differently to US + MBs, in terms of uptake of cell-impermeable dye, reduction in viability and intracellular signalling. Sonoporation is associated with activation of the MAP-kinases p38 and ERK, and increase in the phosphorylation of ribosomal protein S6 together with dephosphorylation of 4E-BP1. This may be a stress-response for cells to survive and repair the cell membrane after sonoporation, resembling cellular responses to electroporation and pore-forming toxins, and are potential drug targets enhancing the efficacy of sonoporation in cancer therapy. As cell types in the tumour microenvironment are more sensitive to sonoporation, further efforts in optimising sonoporation-enhanced therapy should be targeted at the microenvironment.

## Figures and Tables

**Table 1:** US parameters (5 min treatment with US ± microbubbles (MBs)).

Name	Frequency (MHz)	No. of Cycles	Duty Cycle (%)	Pulse Repetition Frequency (kHz)	MI	Intensity	
						I_SPTA_ (mW/cm^2^)	I_SPPA_ (W/cm^2^)
Medium	2.00	80	1.8	22	0.2	50	2.7
High	2.00	160	3.6	22	0.378	358	9.64

## Supplementary Materials

The following are available online at https://www.mdpi.com/1999-4923/12/11/1058/s1, Table S1: Antibody panels for phospho-flow cytometry; Figure S1: Gating strategy of flow cytometric data to identify calcein-positive cells; Figure S2: Gating strategy of flow cytometric data to identify cells stained with AnnexinV and propidium iodide (PI); Figure S3: Time for detachment of cells using cold-trypsinization; Figure S4: Sample processing for phospho-flow cytometry; Figure S5: Gating strategy for identification of each individually stained sample in the barcode (de-barcoding); Figure S6: Supplemental data on viability of cells harvested after 24 h incubation in Petaka G3 LOT®; Figure S7: Increase in ribosomal protein S6 phosphorylation was variable between experiments; Figure S8: Changes in phosphorylation of CREB and PKA was low; Figure S9: Changes in phosphorylation of FAK and Src; Figure S10: Treatment of cancer associated fibroblasts (CAFs) with US + MBs resulted in extensive uptake of the cell impermeable dye (calcein), a large reduction in cell viability and changes in intracellular signalling is observed in the cells that survived sonoporation; Figure S11: Median fluorescence intensity (MFI) of calcein-positive cells; Figure S12: The percentage of cells taking up calcein in HUVEC and HUV-EC-C cell lines; Figure S13: Images of sonoporated cells.
