# Supplementary material for: Low-Intensity Sonoporation-Induced Intracellular Signalling of Pancreatic Cancer Cells, Fibroblasts and Endothelial Cells
Source: Pharmaceutics. 2020 Nov 6;12(11):1058. doi: 10.3390/pharmaceutics12111058 (PMC7694645; doi:10.3390/pharmaceutics12111058)
Supplement: Supplementary file 1 [file pharmaceutics-12-01058-s001.zip › pharmaceutics-966756-supplementary.docx]

Supplementary material：Low-Intensity Sonoporation-Induced Intracellular Signalling of Pancreatic Cancer Cells, Fibroblasts and Endothelial Cells

Ragnhild Haugse, Anika Langer, Elisa Thodesen Murvold, Daniela Elena Costea,
Bjørn Tore Gjertsen, Odd Helge Gilja, Spiros Kotopoulis, Gorka de Ruiz de Garibay and
Emmet McCormack

**Table S1.** Antibody panels for phospho-flow cytometry (all antibodies were obtained from BD BioScience).

| **Protein** | **Phospho-epitope** | **Fluorochrome** |
| --- | --- | --- |
| **Panel 1** |  |  |
| CREB | S133 | Alexa 488 |
| S6 | S235/S235 | Alexa 647 |
| **Panel 2** |  |  |
| ERK | T202/Y204 | Alexa 488 |
| PKA | S99 | Alexa 647 |
| **Panel 3** |  |  |
| FAK | S910 | Alexa 488 |
| STAT3 | S727 | Alexa 647 |
| **Panel 4** |  |  |
| S6 | S240 | Alexa 488 |
| PKA | S114 | Alexa 647 |
| **Panel 5** |  |  |
| p38 | T180/Y182 | Alexa 488 |
| Akt | S473 | Alexa 647 |
| **Panel 6** |  |  |
| Src | Y418 | Alexa 488 |
| 4E-BP1 | T36/45 | Alexa 647 |


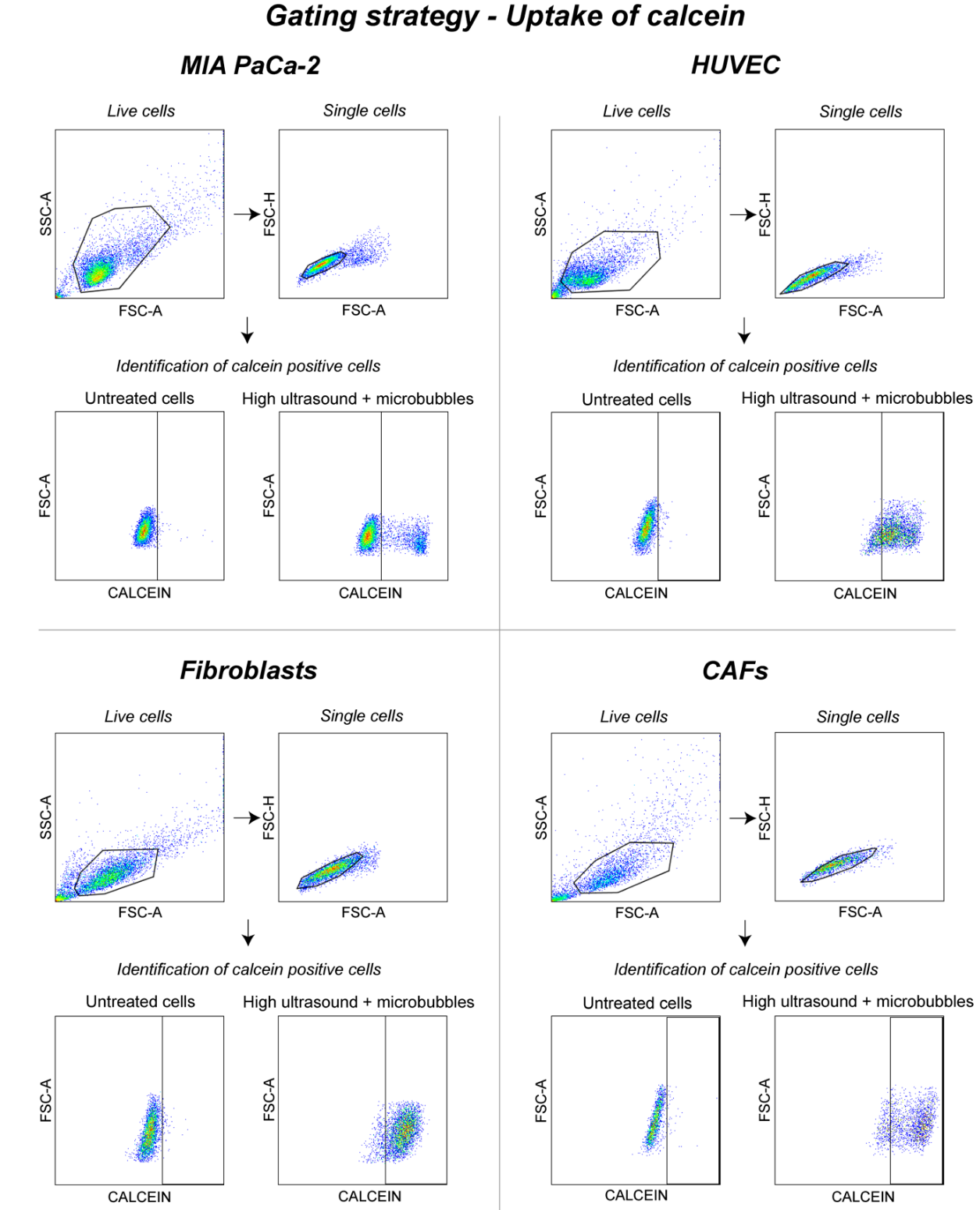


**Figure S1.** Gating strategy of flow cytometric data to identify calcein-positive cells.


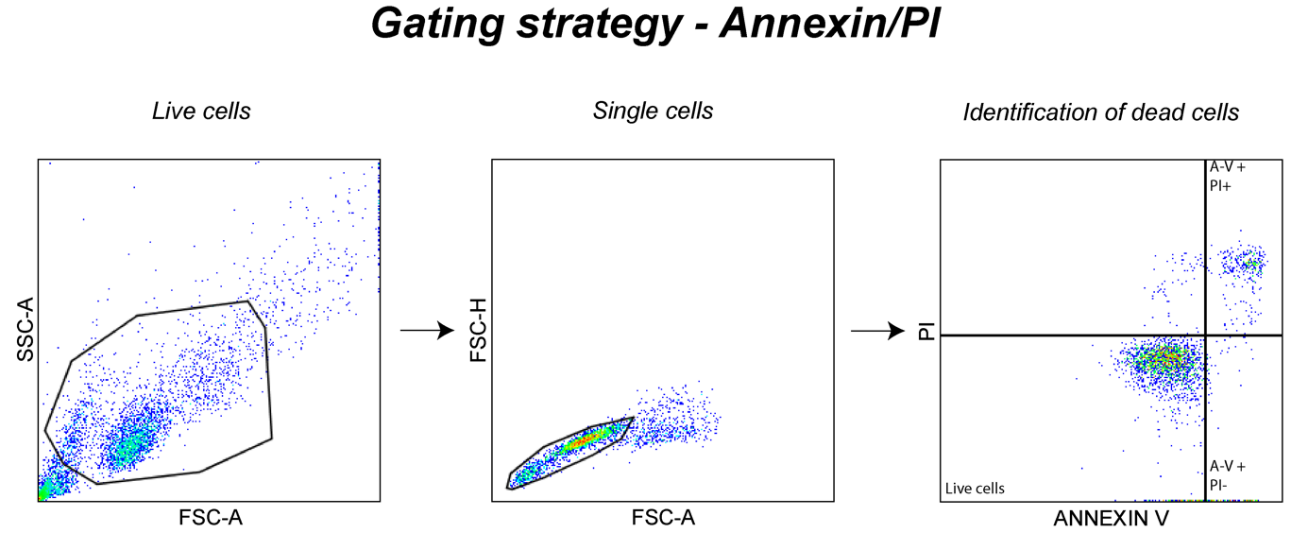


**Figure S2.** Gating strategy of flow cytometric data to identify cells stained with AnnexinV and propidium iodide (PI) (scatter plots for MIA PaCa-2 used as example; the same gating strategy was used for all cell lines).


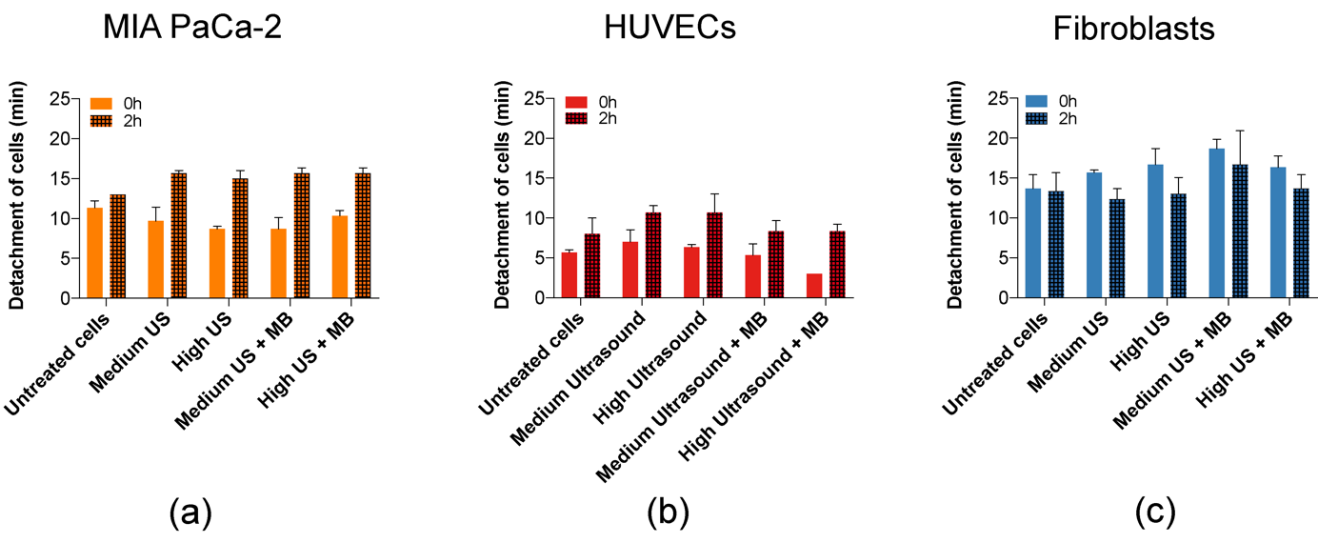


**Figure S3.** Time for detachment of cells using cold-trypsinization (min). Mean ± SEM.


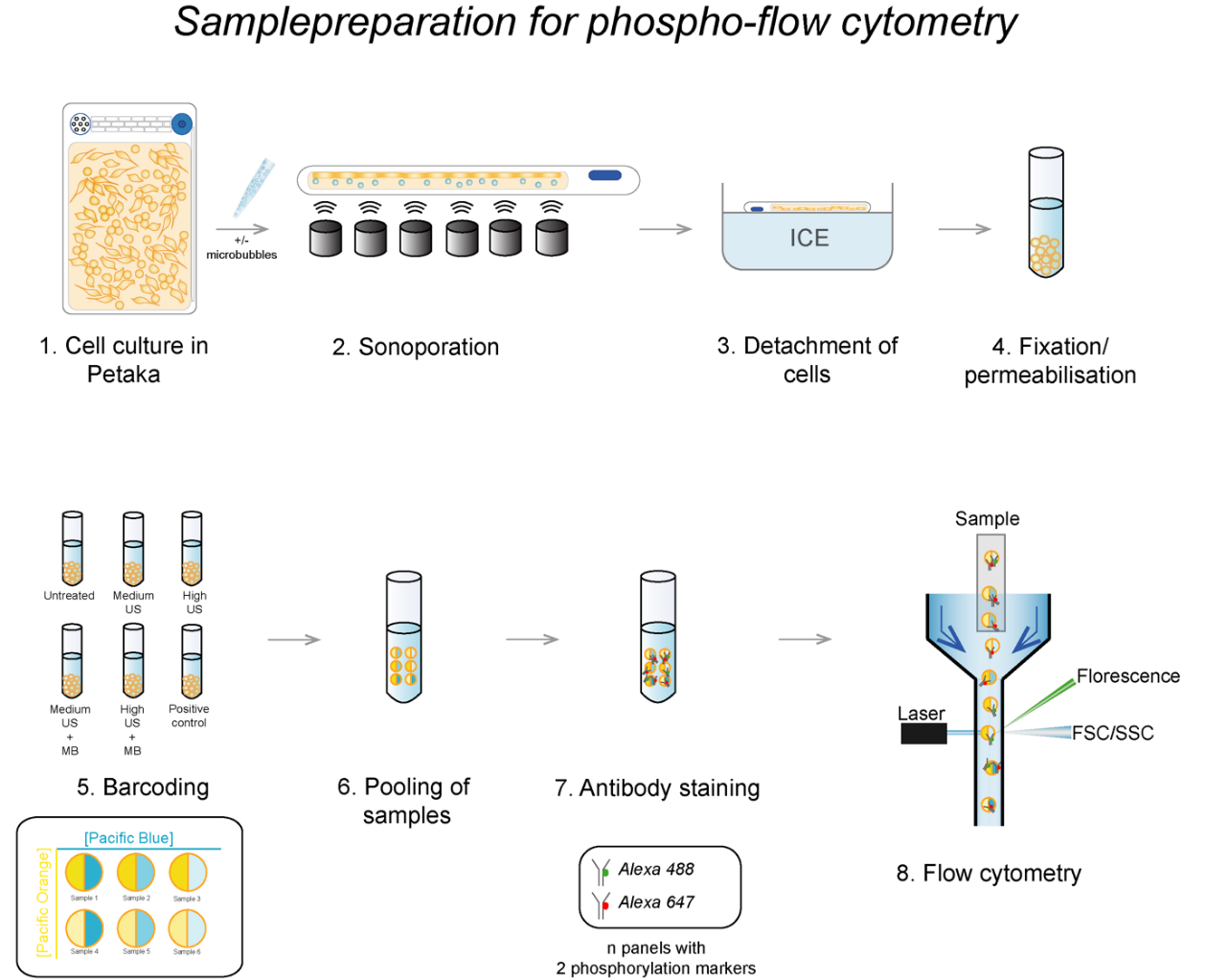


**Figure S4.** Sample processing for phospho-flow cytometry; Cells were cultured in Petaka G3 LOT^®^ (1) before addition of MB and US exposure (2). After sonoporation the cells were detached on ice using the cold-trypsin method (3), fixed and permeabilized (4), barcoded (5), samples were pooled (6), stained with phosphorylation specific antibodies (7) and analyzed by flow cytometry (8). Barcoding of cells allows for reduced analytical variation and increases throughput. All samples harvested at a certain timepoint (immediately or 2 h after sonoporation) at medium and high ultrasound intensity were barcoded and pooled in to one sample per timepoint. This allow for normalization of all treated samples to untreated cells in the same barcode during data analysis. Pooled samples were split in tubes to be stained with panels consisting of 2 phosphospecific antibodies conjugated to respectively Alexa 488 and Alexa 647.


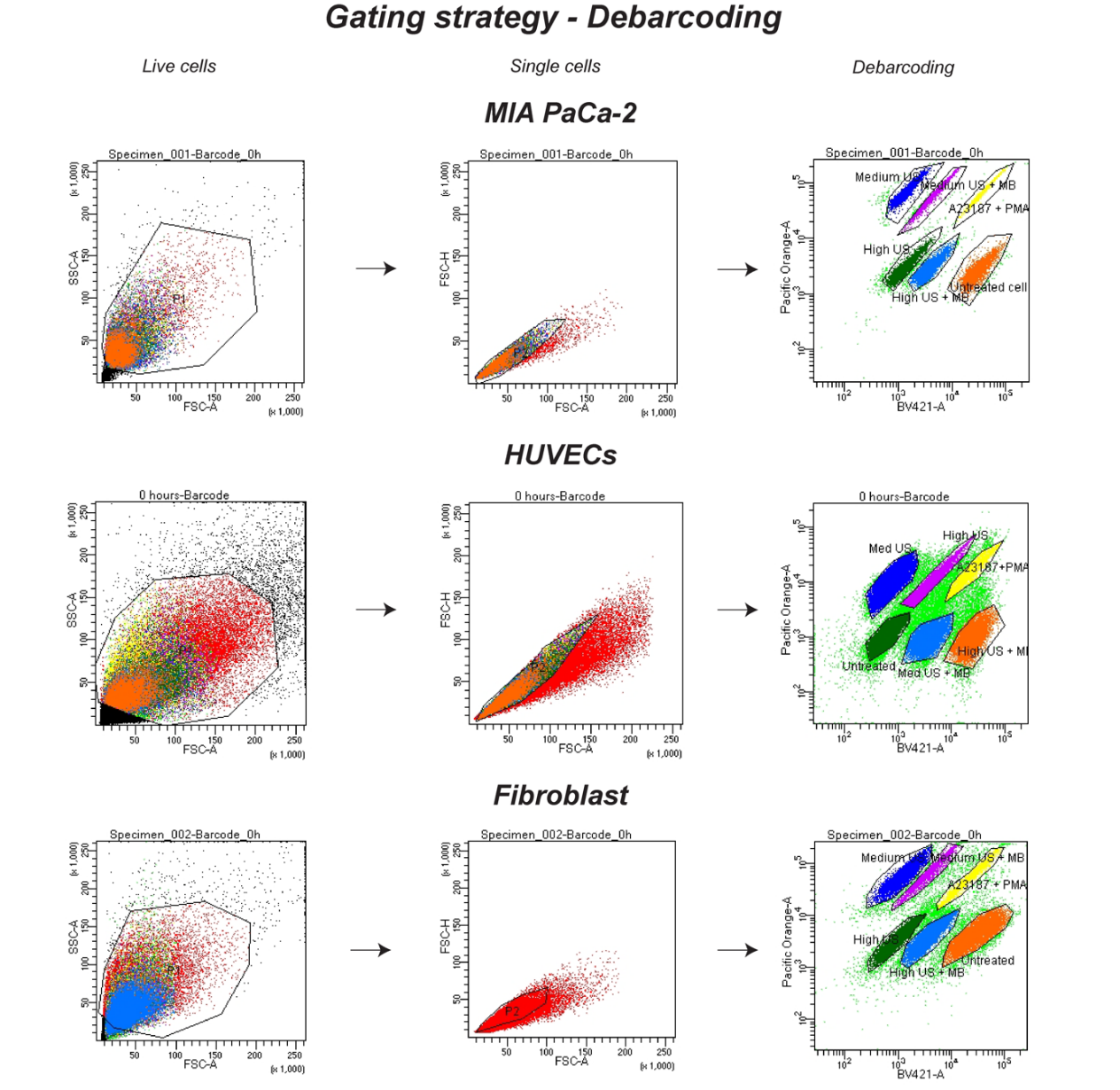


**Figure S5.** Gating strategy for identification of each individually stained sample in the barcode (de-barcoding). One pooled sample (untreated cells, medium US, high US, positive control, medium US + MB, high US + MB) was barcoded per timepoint after sonoporation.


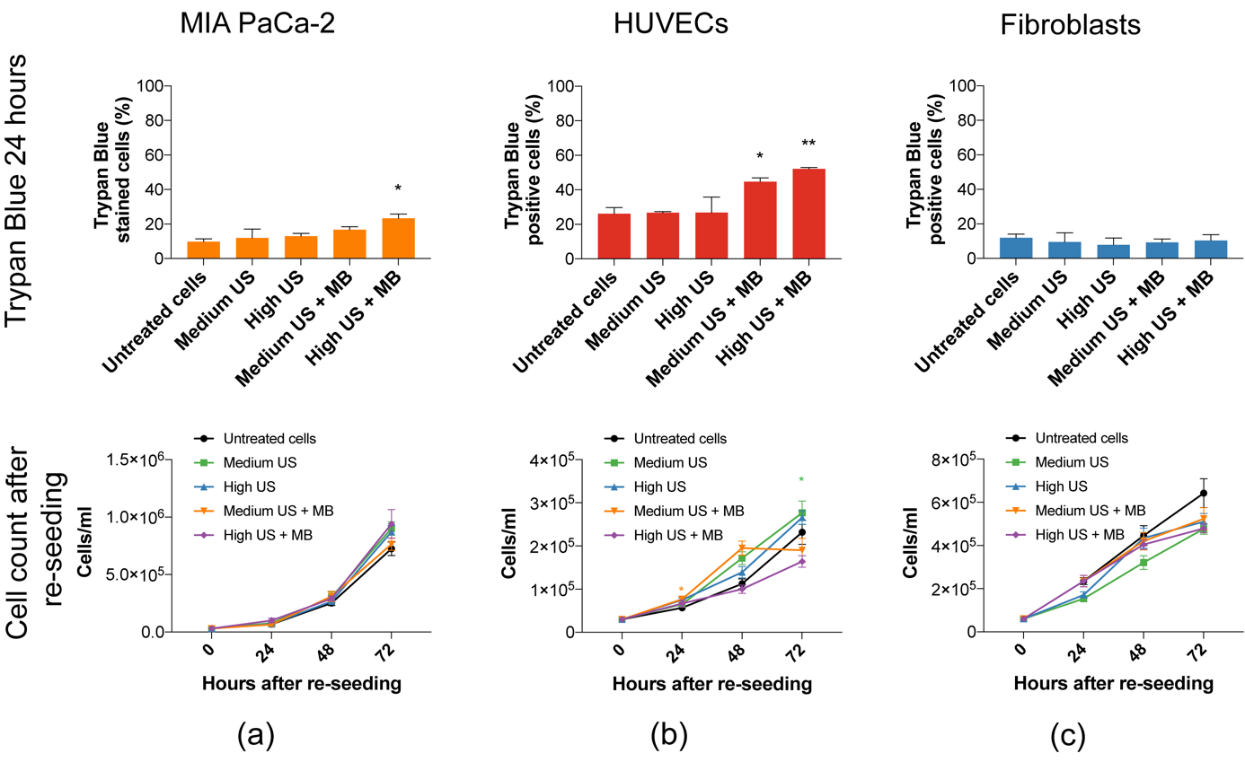


**Figure S6.** Supplemental data on viability of cells harvested after 24 h incubation in Petaka G3 LOT^®.^ **(a)** The number of Trypan Blue stained MIA PaCa-2 cells was increased by treatment with High US + MB. The cell count after re-seeding was not affected by US + MB **(b)** The number of Trypan Blue stained HUVECs was increased by treatment with US + MB. The cell count after re-seeding was initially higher in cells treated with Medium US + MB, but lower after 72 h (ns). Cell count was lower for cells treated with high US + MB at all time points (ns). Cells treated with Medium US experienced largest increase in cell count (*p* < 0.05) **(c)** No differences in percentage Tryphan Blue stained cells was observed in fibroblasts. The cell count after re-seeding was initially higher in cells treated with US + MB, but lower after 72 h (ns). Mean ± SEM; **p* < 0.05, ***p* < 0.01 (treated vs untreated cells).


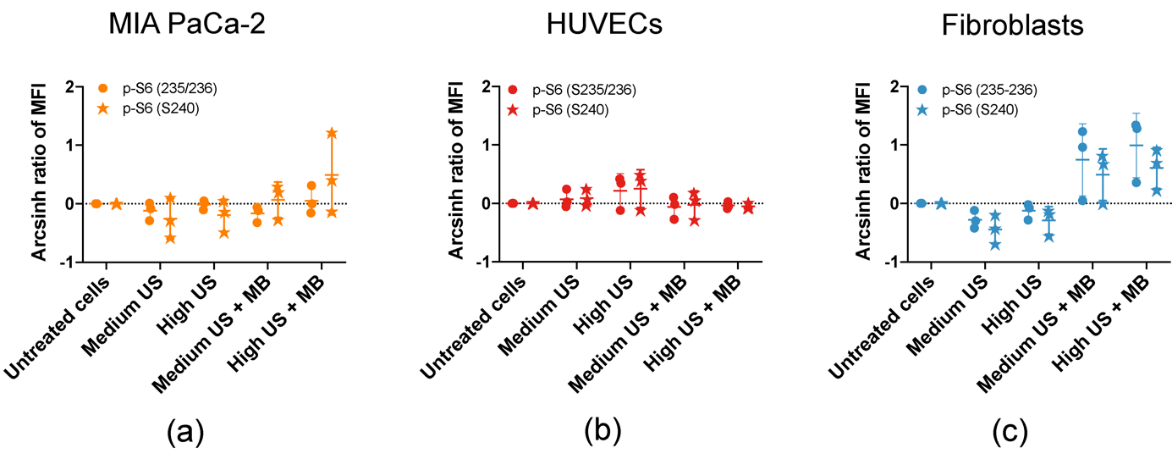


**Figure 7.** Increase in ribosomal protein S6 phosphorylation was variable between experiments. The increase in phosphorylation of ribosomal protein S6 was not statistically significant, most likely due to the experimental variation **(a)** MIA PaCa-2 **(b)** HUVECs **(c)** Fibroblasts.


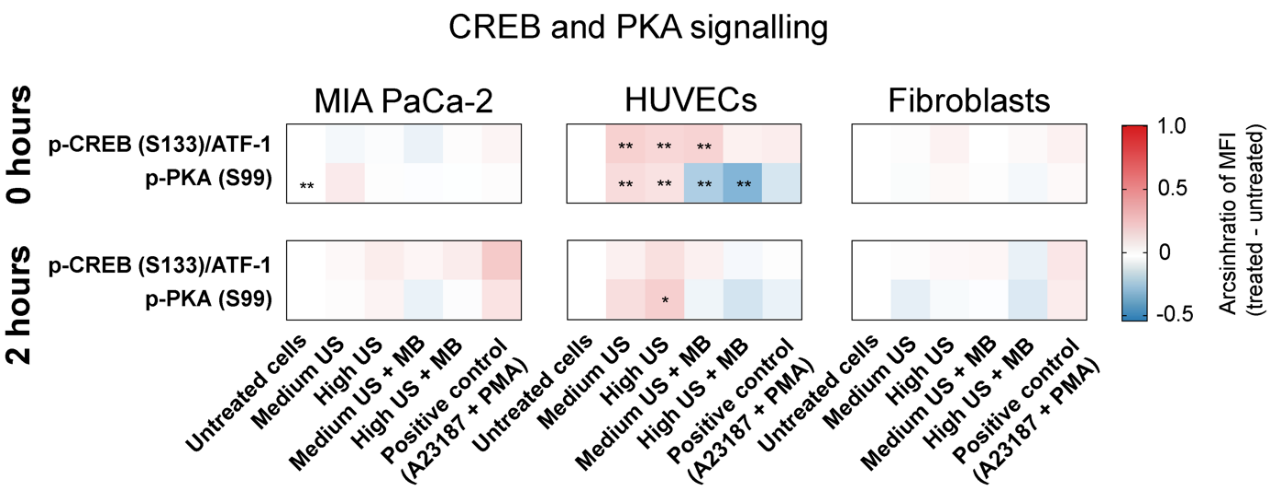


**Figure S8.** Changes in phosphorylation of CREB and PKA was low. Since these proteins were not reliably activated by the positive control A23187 (Ca^2+^ ionophore) + PMA (phorbol myristate acetate = PKC activator) results are inconclusive. Mean ± SEM; **p* < 0.05, ***p* < 0.01 (treated vs untreated cells).


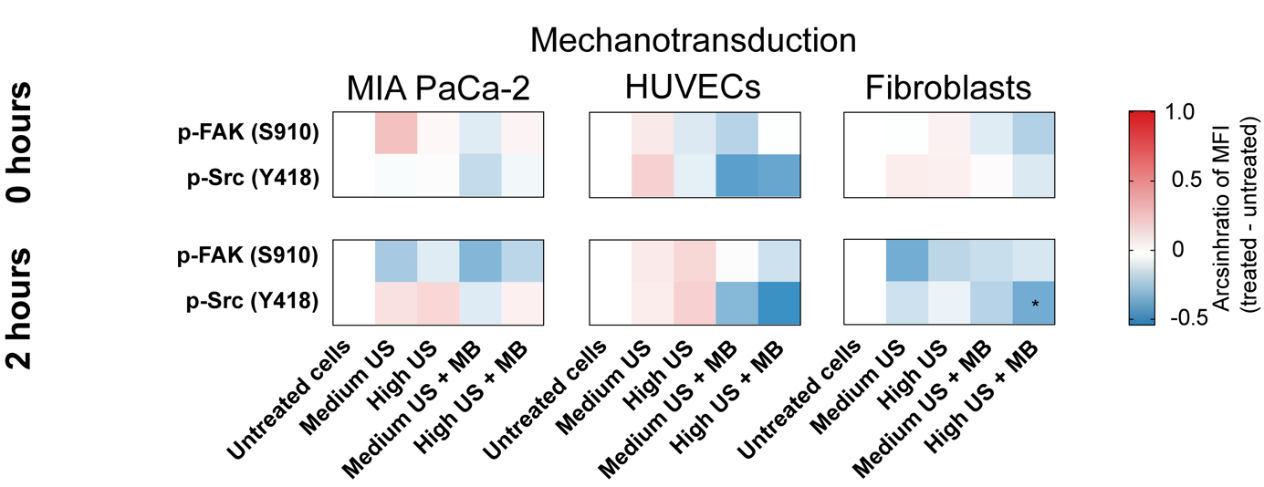


**Figure S9.** Changes in phosphorylation of FAK and Src. Mean ± SEM; * p < 0.05 (treated vs untreated cells).


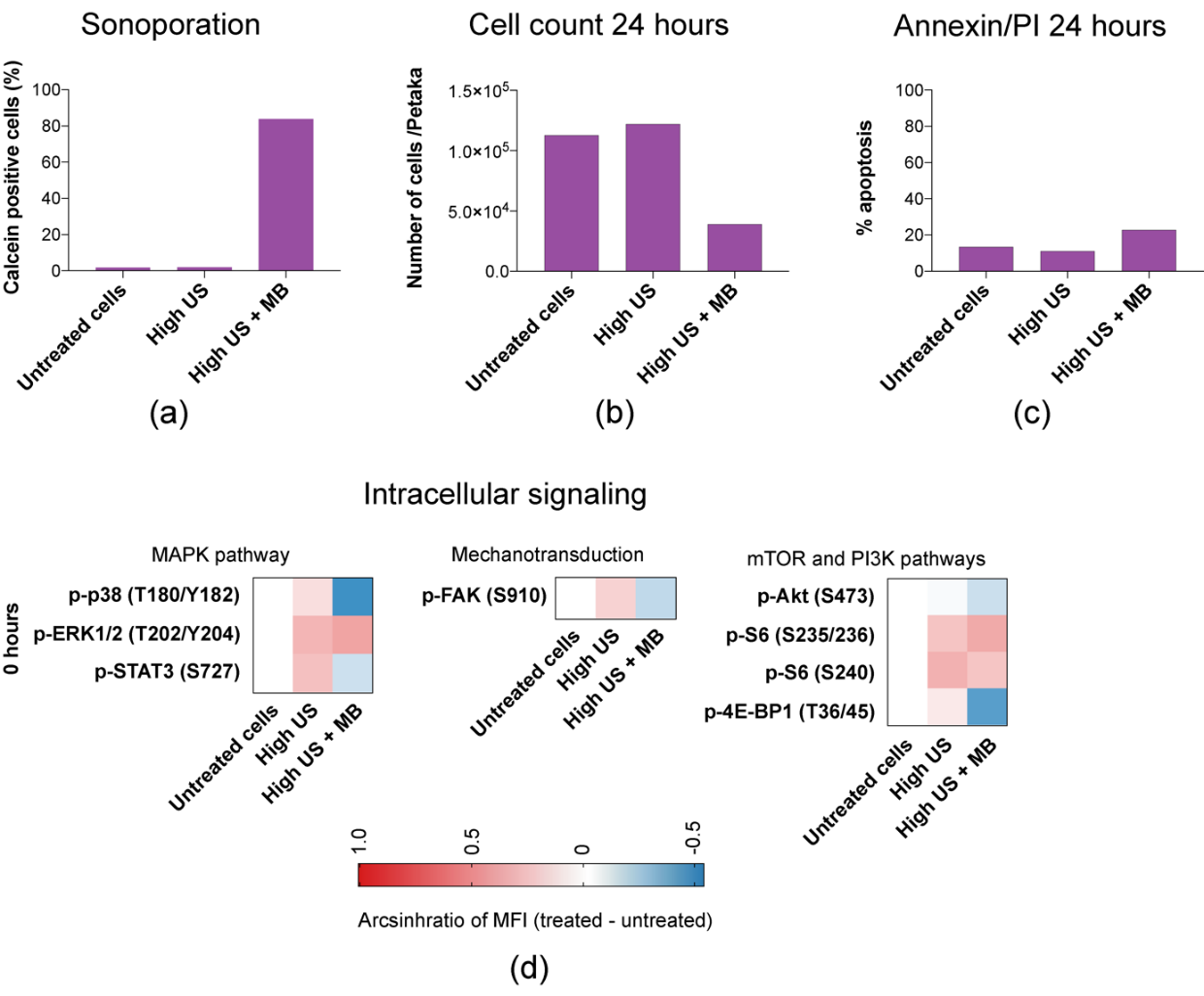


**Figure S10.** Treatment of cancer associated fibroblasts (CAFs) with US + MBs resulted in extensive uptake of the cell impermeable dye (calcein), a large reduction in cell viability and changes in intracellular signalling is observed in the cells that survived sonoporation **(a)** Percentage of cells taking up calcein, a cell-impermeable dye, was increased to ≥ 80% by sonoporation. **(b)** Cell count was reduced 24 h post sonoporation and **(c)** percentage of apoptotic cells was increased 24 h post sonoporation. **(d)** Heatmaps displaying changes in phosphorylation status (shown as arcsinh ratio) of the chosen range of proteins in response to treatment with US with and without Sonazoid^TM^ MBs immediately (0 h) after sonoporation, showing activation of ERK1/2 and ribosomal protein S6 and deactivation of p38 and 4E-BP1. (n=1).


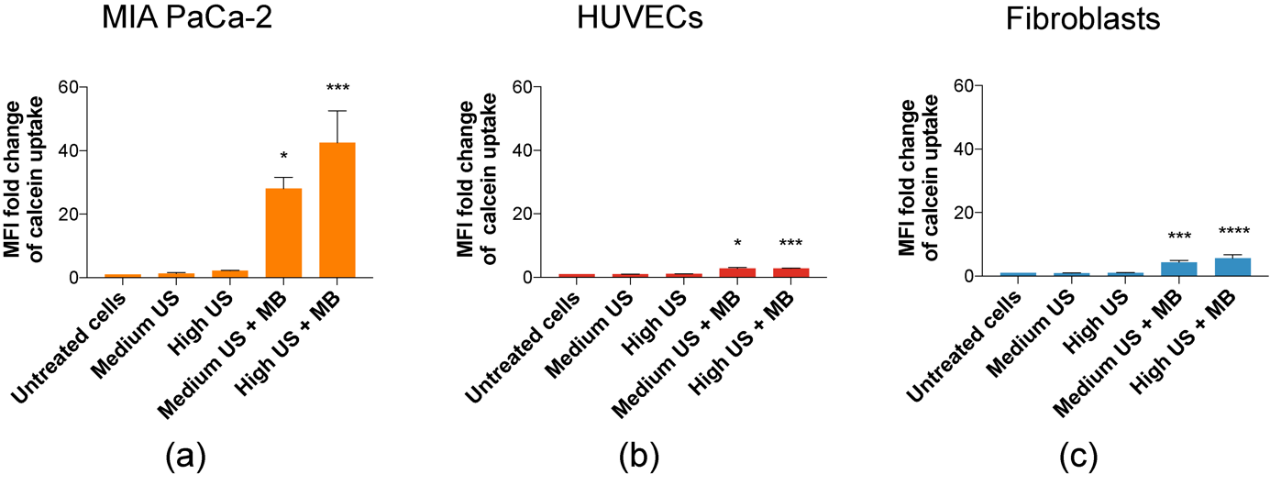


**Figure S11.** Median fluorescence intensity (MFI) of calcein-positive cells. Addition of microbubbles was important for amount of calcein taken up by the cells. The largest MFI was detected in **(a)** MIA PaCa-2 compared to **(b)** HUVECs and **(c)** Fibroblasts. Mean ± SEM; **p* < 0.05, ****p* < 0.001, *****p* < 0.0001.


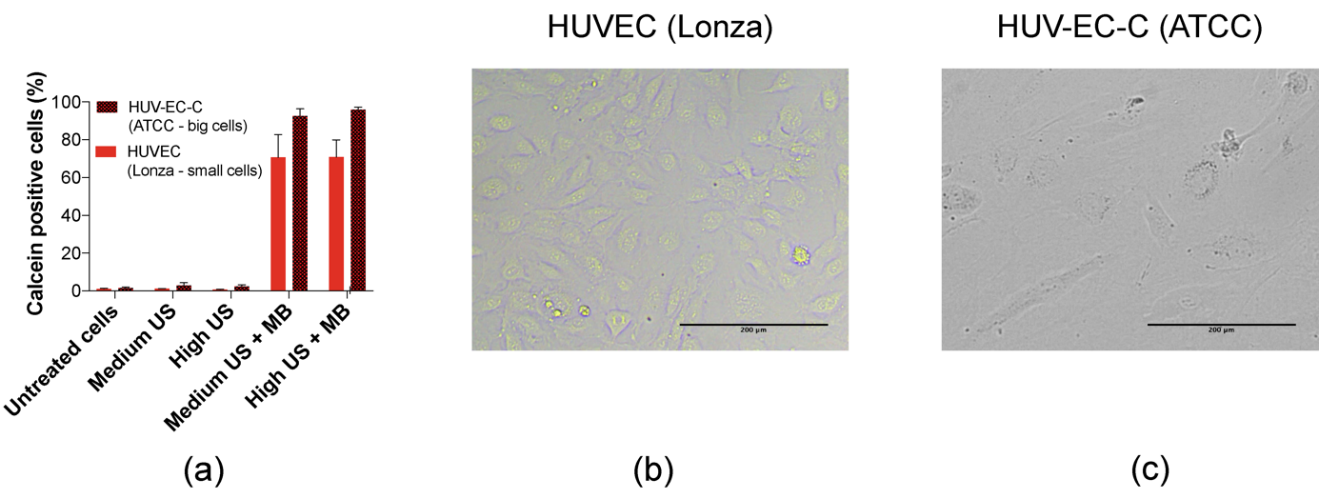


**Figure S12.** The percentage of cells taking up calcein in HUVEC and HUV-EC-C cell lines **(a)** Percentage of HUVEC cells taking up calcein: comparison between HUVEC (Lonza–small cells) vs HUV-EC-C (ATCC, large cells). No significant differences (Mean ± SEM) **(b)** Image of HUVEC (Lonza – small cells) **(c)** Image of HUV-EC-C (ATCC, large cells) including scale bar.


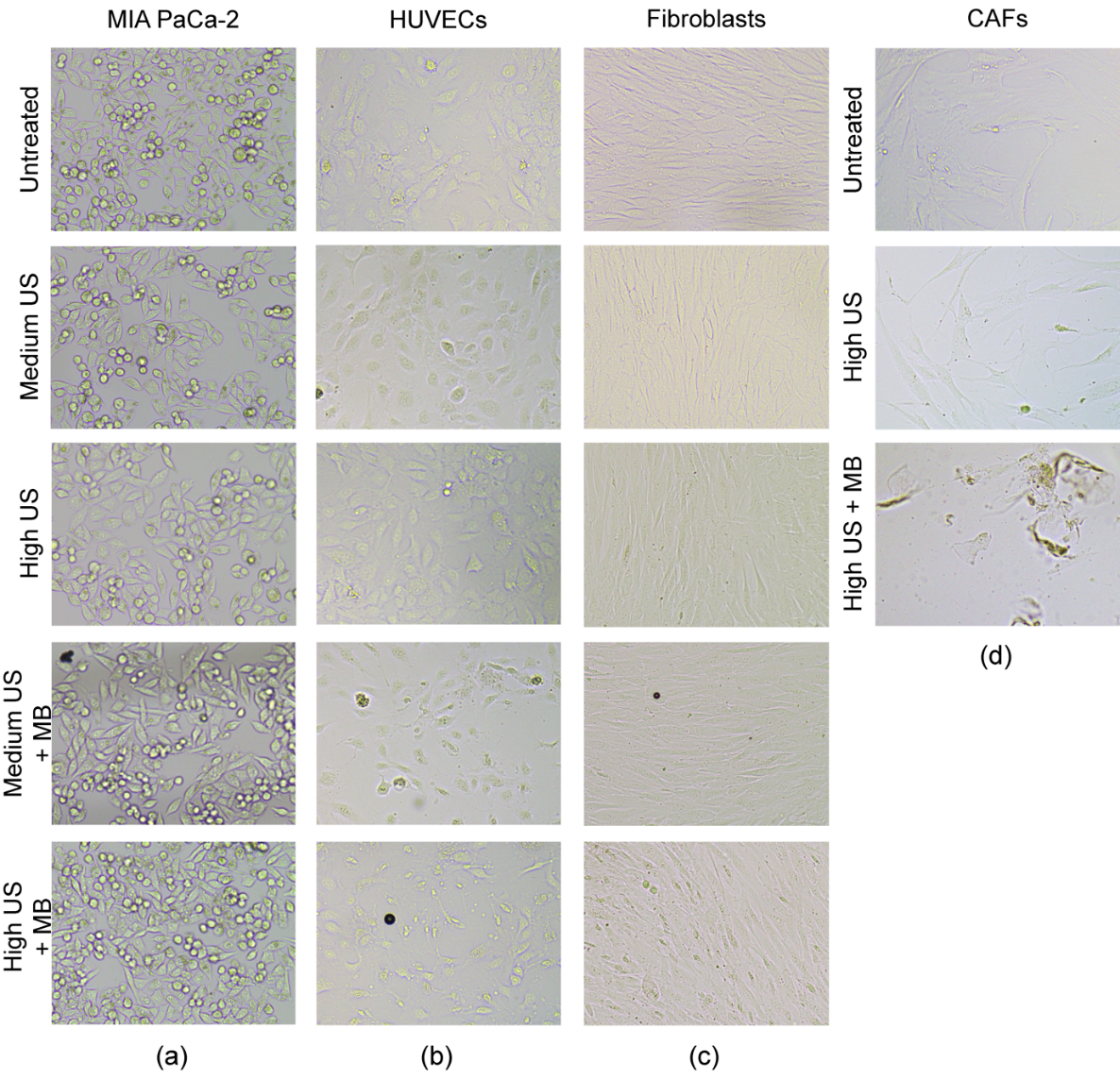


**Figure S13.** Images of sonoporated cells **(a)** Miapaca **(b)** HUVECs **(c)** Fibroblasts **(d)** CAFs.
